# Sudden-Onset Paraplegia: A Unique Presentation of Acute Aortic Dissection (AD)

**DOI:** 10.7759/cureus.33389

**Published:** 2023-01-05

**Authors:** Monica R Campa, Brook Danboise, John D Cambron

**Affiliations:** 1 Emergency Medicine, Christus Health/Texas A&M University School of Medicine, Corpus Christi, USA

**Keywords:** emergency diagnosis, cardiovascular injury, resuscitation, aortic dissection, emergency medicine

## Abstract

Aortic dissection (AD) is a rare but deadly diagnosis that emergency medicine physicians must consider in a wide variety of patient presentations. This case report describes a 42-year-old male bull rider who developed acute-onset bilateral lower extremity paralysis and loss of sensation. He was later found to have a type A Stanford AD during his emergency department evaluation.

## Introduction

Acute aortic dissection (AD) is an uncommon disorder with an incidence of 4.4 per 100,000 person years [1-5]. Emergency medicine physicians know AD to be a challenging presentation as they are rare and difficult to diagnose due to non-specific presentations [1-4]. As many as one-third of the patients do not have AD diagnosed upon initial presentation with delays and failure to recognize its signs and symptoms contributing to its high mortality [1-4].

Research continues to demonstrate that the most common presenting symptom of AD is severe pain [1-4,6]. Although it is classically taught that focal neurologic deficits may be present, the most common neurologic symptom tracked in literature tends to be syncope [2-4]. In one study comparing patients with AD to those with chest or back pain of other etiologies, all patients who presented with focal neurologic signs ended up being diagnosed with AD. However, in that study of 250 patients, only 11 had paraparesis or hemiplegia [6]. This suggests that focal neurologic deficits are an important presentation for physicians in the emergency department to consider for dissection.

In the Stanford classification system, dissections occurring in the ascending aorta are classified as type A dissections. Dissections occurring in the descending aorta are classified as type B dissections [1]. Stanford type A dissections on average carry a mortality rate of approximately 20% [1]. When diagnosed consultation with a cardio-thoracic surgeon and emergency surgical intervention is warranted [1-4]. If the dissection involves only the descending aorta (type B), medical treatment is indicated and surgery usually is not recommended [1]. Regardless of the type of dissection, the initial treatment goals are to control the tear via reduction of systolic blood pressure and determine whether surgically repairing the tear would benefit the patient [1-5].

## Case presentation

A 42-year-old male with a history of aortic root dilation and methamphetamine use presented to our emergency department for sudden onset of paralysis and numbness from the waist down. When he awoke that morning, he experienced chest pain with radiation into his abdomen and back. When he tried to get up from bed, he was unable to do so due to complete paralysis of both lower extremities. On the arrival of emergency medical services (EMS), the patient was noted to be bradycardic with a heart rate of 40 and hypotensive with initial systolic blood pressure of 80. There were no palpable pulses in his bilateral feet initially. He was given atropine in route via EMS for treatment of bradycardia. Aortic root dilation was incidentally detected via CT scan of chest in 2013. Aortic root measured 4.8cm at that time; the patient unfortunately did not follow up with cardio-thoracic surgery referral and was lost to follow up.

On arrival at our emergency department, the patient was alert and oriented. Initial vitals included a heart rate of 93 and a blood pressure of 122/45. He described 10/10 sharp, diffuse abdominal pain with radiation into his back as well as continued lower extremity paralysis. He did note that two to five days ago, he went bull riding but did not recall any significant injury. Physical exam was remarkable for complete loss of sensation and motor function in his bilateral lower extremities. His dorsalis pedis and posterior tibial pulses were initially not palpable. However, on return from an urgent CT scan his pulses were noted to be palpable.

Initial laboratory workup revealed a hemoglobin of 15.5 g/dL, and complete metabolic profile (CMP) and initial troponin were unremarkable. Coagulation studies revealed prolonged prothrombin time (PT) at 13.9 and partial PT (PTT) at 53. Urine drug screen was positive for amphetamines and cannabinoids. His rapid COVID-19 was positive.

**Figure 1:**
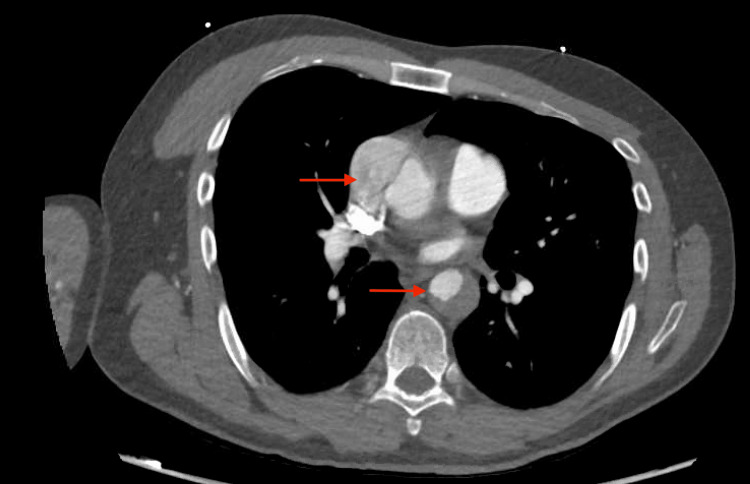
Dissection involving ascending/descending aorta

**Figure 2:**
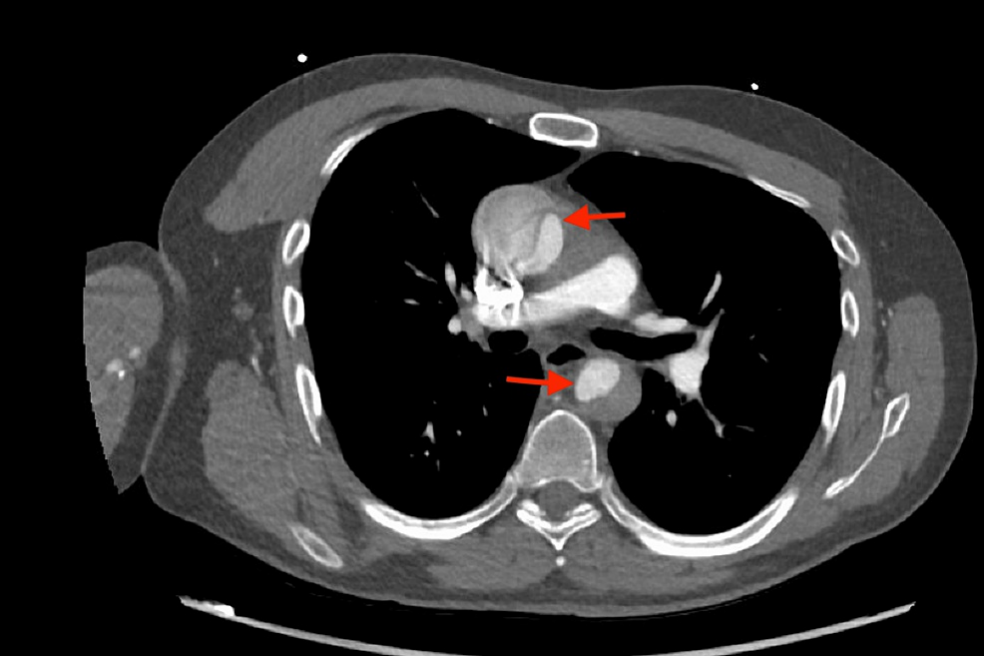
Continued AD AD: Aortic dissection

**Figure 3:**
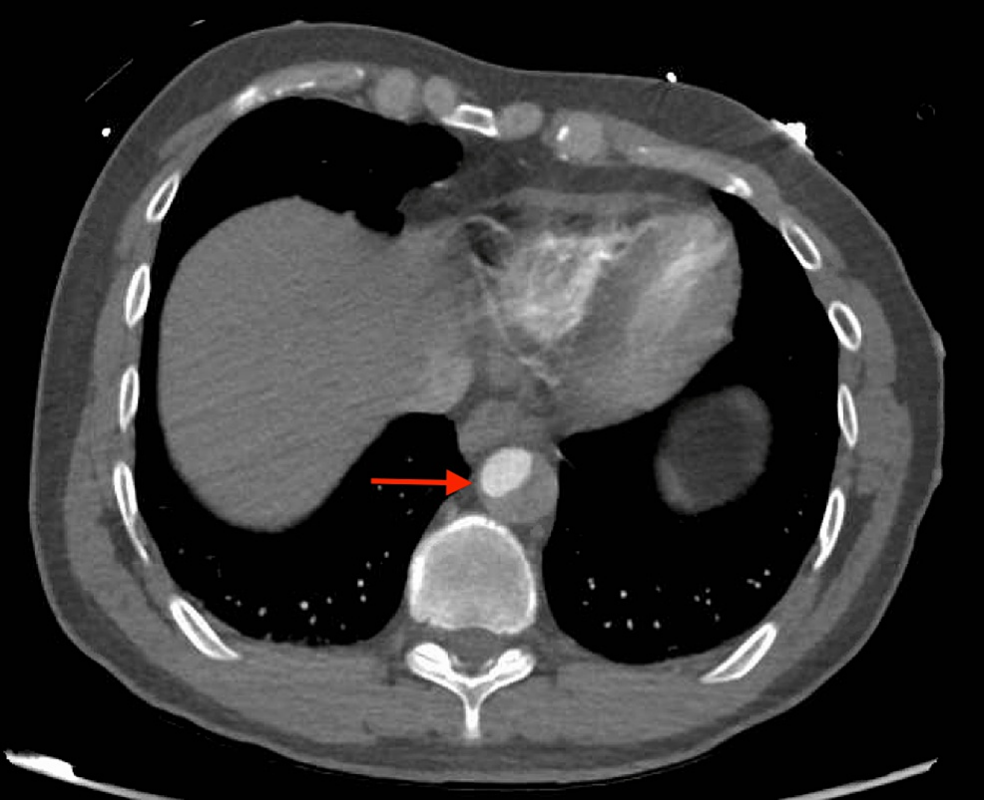
AD involving descending aorta AD: Aortic dissection

CT angiography of chest/abdomen/pelvis demonstrated extensive AD with type A Stanford dissection involving both the ascending and descending aorta (Figures 1,2,3). The dissection extended from the common carotid arteries to the iliac arteries.

Due to the complexity of the patient's dissection, he required transfer to a higher level of care. Unfortunately, there was a delay in transfer of two days due to multiple hospitals declining due to either capacity issues or lack of specialist availability. The patient’s positive COVID-19 status also complicated his transfer. While awaiting transfer, the patient was medically managed in the emergency department in conjunction with intensivists. He was treated with an esmolol infusion with a systolic blood pressure goal of 100-120 and heart rate less than 60. Pain was controlled with intermittent fentanyl and morphine boluses.

Labs monitored throughout his extended stay in the emergency department showed development of a leukocytosis, and his hemoglobin dropped by 2 g/dL. His CMP demonstrated decreased renal perfusion with blood urea nitrogen (BUN)/creatinine going from 9/1.4 to 29/1.5. He developed a lactic acidosis of up to 6.2 consistent with ischemia. The patient’s troponin also trended up to 1.046 after initially being negative at presentation. Multiple electrocardiograms were obtained throughout patient stay that did not demonstrate acute ischemic findings.

After almost 48 hours in our emergency department, the patient was transferred to a higher level of care where an available cardio-thoracic team intervened. He underwent an arch and Bentall repair, which can be quickly summarized as a surgical repair of an ascending aortic or aortic root aneurysm in combination with aortic valve disease [7]. Less commonly, it is used to repair AD affecting the aortic root and valve [7]. He survived the procedure and was later extubated in the ICU. Postoperatively, he developed complete heart block requiring pacemaker placement, renal insufficiency, spinal cord ischemia, pericardial effusion requiring pericardial window, and a sternal wound infection. He did not regain lower extremity function.

## Discussion

Our patient presented to the emergency department with acute pain and sudden-onset bilateral lower extremity loss of sensation and motor function related to an extensive AD. His symptoms appeared to be caused by anterior versus complete spinal cord ischemia. Given the fact that his sensation was also compromised, he may have been experiencing complete transverse myelopathy due to complete ischemia of his spinal cord. An MRI was ordered but unfortunately, it was not obtained during his stay in our emergency department due to initial patient refusal.

Very few cases have been reported in which an AD presents with acute lower extremity paraparesis as the major initial complaint [8-11]. Our patient surely experienced acute spinal cord ischemia due to the injury of his thoracic aorta compromising blood flow to his spinal cord. It is entirely possible that the artery of Adamkiewicz was involved in the aortic injury [12]. The artery of Adamkiewicz supplies arterial blood to the spinal cord from T8 to the conus medullaris [12]. Since this presentation is so rare, AD is not always at the top of the list of differentials that clinicians tend to consider. Literature already demonstrates that it is imperative for emergency physicians to maintain a high index of suspicion for the uncommon diagnosis of AD. Our case report additionally highlights the need for clinicians to consider AD in rare patients that present with unique symptoms concerning for anterior or complete spinal cord ischemia.

## Conclusions

AD is a rare presentation that carries significant risk of morbidity and mortality. All practicing emergency physicians should be adept at detecting and managing the patient with an acute AD. Given the dire consequences of delayed intervention associated with an AD it is important that unique symptoms be presented to medical providers. It is our hope that this case will aid others in being able to diagnose and manage this deadly condition in an expedited manner.
